# Polyploid plant genomes complexity and the challenges of sequencing

**DOI:** 10.1007/s00425-026-04952-w

**Published:** 2026-03-09

**Authors:** Aaron W. Anderson, Emidio Albertini, Daniele Rosellini

**Affiliations:** Dipartimento di Scienze Agrarie, Alimentari e Ambientali, Università Degli Studi di Perugia, Borgo XX Giugno 74, 06121 Perugia, Italy

**Keywords:** Polyploidy, Genome sequencing, Genome assembly, Whole-genome duplication, Neopolyploidy, Plant breeding, *Medicago sativa*

## Abstract

**Main conclusion:**

Whole-genome duplication is an important evolutionary mechanism for many agriculturally important plants. We discuss selected polyploid genomic studies, limitations, and practical applications in plant breeding.

**Abstract:**

Polyploids are highly represented among agriculturally important plant species. Understanding how plant genomes change in response to whole-genome duplication is important for streamlining the use of diploid germplasm in polyploid breeding programs to introduce new alleles, genes, or desirable traits. The complexity of polyploid genomes stemming from their diverse evolutionary histories poses challenges for assembling high-quality, haplotype-resolved genome sequences, a necessary step for optimizing plant breeding efforts. In this review, we examined genomic studies that yielded high-quality reference genomes through novel approaches in various polyploid crop species. Examples include using references of progenitor species in peanut and blueberry, tackling the mixed-ploidy levels of sugarcane and dealing with species complexes in wheat and alfalfa. We also highlighted the new and innovative approaches to polyploid genome sequencing used in these studies as well as others. These methods and tools can be especially useful in species where genomic studies are not advanced, to provide insights on adapting techniques in other polyploid species to create more refined genomic studies crop improvement.

## Polyploidy in plants

Polyploidy is the presence of more than two genomes in a cell or an organism. It arises due to whole-genome duplication (WGD), an important evolutionary mechanism for plants (Salman-Minkov et al. 2016). Roughly 35% of all extant flowering plants are considered recent polyploids (Rice et al. 2019) though it has been estimated that the actual number may be as high as 70% (Sattler et al. 2016). While the true number of polyploids is debated, cytogenetic and genomic studies have shown that WGD has been a recurrent and important aspect of plant speciation throughout evolutionary history, and it is now accepted that all angiosperms have experienced multiple WGDs during their evolution (Jiao et al. 2011; Salman-Minkov et al. 2016).

Polyploid plants have been classified into two main types: autopolyploids and allopolyploids. Although this historical classification is debated and does not capture the complexities of polyploids origins and inheritance (Twyford et al. 2025 and references therein), it is still useful when discussing genome structure and sequencing. Autopolyploids arise from a single progenitor species that underwent WGD (Bretagnolle And Thompson 1995). Allopolyploids arise from two or more progenitor species that have combined into a single species (Gaeta And Chris Pires 2010). A third, less common situation is that of species classified as auto-allopolyploid, where allopolyploids have undergone a duplication event of one or all the genomes they contained, as described in sugarcane (Shearman et al. 2022). There are nuances within each ploidy type as well, regarding formation, reproduction, and function; multiple polyploidy events may arise within a taxon, from which different species may evolve (Adams And Wendel 2005). Early generations of polyploids after WGD are referred to as neopolyploids (Ramsey And Schemske 2002). Neopolyploids may fail to establish, outcompete their diploid progenitors, causing their complete erasure, or exist in mixed-ploidy populations. Plants are able to combine ploidy types, making any combination of duplicated genomes from a single progenitor species, or mixed genomes from multiple progenitor species, so that neopolyploids can exists at many stages and ploidy levels simultaneously (Mata et al. 2023; Nadon And Jackson 2020).

Specific nomenclature is used to describe the origins of a polyploid, as well as the overall ploidy level. The letters “*n*” and “*x*” are used to describe the ploidy state of a species: “*n*” refers to the gametic (n) or somatic (2n) chromosome number of the species, irrespective of ploidy; “*x*” denotes how many sets of chromosomes are contained in a cell (Van De Peer 2023). In diploid species, typically, 2n = 2x. In polyploids, however, 2n can equal 4x (tetraploid), 6x (hexaploidy), etc., depending on the number of chromosome sets. The genomes within a polyploid species are denoted with letters: for example, “AAAA” for an autotetraploid species and “AABB” for an allotetraploid species (Van De Peer 2023). In the case of autotetraploid alfalfa (*Medicago sativa L.*), the chromosome number is denoted as 2n = 4x = 32, AAAA, meaning that a somatic cell contains 4 chromosome sets, each made of 8 chromosomes, and each chromosome is present in 4 copies. Nomenclature is standardized so that the genomes contributing to a polyploid are associated with a defined parental diploid species. This can also be used to track genomes in species where multiple allopolyploidization events have happened, so two allopolyploids may share a progenitor species, such as *Triticum* (Van De Peer 2023).

Table 1 lists all the species discussed in this review, providing common names, identifying species complex, relevant ploidy levels and class, and a primary source for the information. This is not an exhaustive list of polyploid species, but a representative sample used for the discussion of genome assembly and the impacts of WGD in this review. Further, this review aims to draw parallels between polyploid studies in a breadth of species with the overall goal of encouraging the use or adaptations of methods developed in more deeply studied species.

**Table 1:** A comprehensive list of common names, scientific names, species complex, sub species, ploidy classification and level of all species discussed in this review with primary source noted

Common name	Scientific name	Ploidy class(s)	Ploidy level(s)	Source
Cultivated Alfalfa	*Medicago* complex	auto	2x, 4x	Small (2011)
	* M. sativa* ssp. *sative* L.	auto	4x	
	* M. sativa* ssp. *falcata*	auto	2x, 4x	
	* M. sativa* ssp. *caerulea*		2x	
	* M. sativa* ssp. *hemicycla*		2x	
	* M. sativa* ssp. x *varia*	auto	4x	
	* M. prostrata*		2x	
	* M. truncatula*		2x	
	* M. scutellata*		2x	
Brasica	*Brassica*	allo	2x, 4x	Li et al. (2017)
Field mustard	* B. rapa*		2x	
Wild cabbage	* B. oleracea*		2x	
Black mustard	* B. nigra*		2x	
Rapeseed	* B. napus*	allo	4x	
Chinese mustard	* B. juncea*	allo	4x	
Ethiopian mustard	* B. carinata*	allo	4x	
Salsifies	*Tragopogon*	allo	2x, 4x	Soltis et al. (2004)
	* T. mirus* Ownbey	allo	4x	
	* T. miscellus* Ownbey		4x	
	* T. dubius* Scop.		2x	
	* T. pratensis* L.		2x	
	* T. porrifolius* L.		2x	
Cultivated banana	*Musa acuminata* complex	auto, allo	2x, 3x, 4x	Li et al. (2024)
	* M. acuminate* ssp. *banskii*		2x	
	* M. acuminate* ssp. *malaccensis*		2x	
	* M. acuminate* ssp. *zebrina*		2x	
Wheat	*Triticum x Aegilops* complex	allo	2x, 4x, 6x	Matsuoka et al. (2014)
Bread wheat	* T. aestivum*	allo	6x	
Taush’s goatgrass	* A. tauschii*		2x	
Durum wheat	* T. turidum* ssp. *durum*	allo	4x	
Cultivated emmer	*T. turidum* ssp. *dicoccon*	allo	4x	
Wild emmer	*T. turidum* ssp. *dicoccoides*	allo	4x	
Peanut	*Arachis hypogaea*	allo	2x, 4x	Moretzsohn et al., 2013
	* A. thaliana*		2x	
	* A. arenosa*		2x	
Paspalum	*Paspalum*	auto, allo	2x-16x	
Bahiagrass	* P. intermedium*	auto	2x, 4x	Karunarathne et al. (2020)
Red paspalum	* P. rufum*	auto	2x, 4x	Soliman et al. (2021)
Arabidopsis	Arabidopsis	auto, allo	2x, 4x, 6x, 8x	J. Wang et al. (2006)
	* A. thaliana*	allo	2x, 4x	
	* A. arenosa*	auto, allo	2x, 4x	
Rice	*Oryza sativa*	allo*	2x, 4x	Han et al. (2025)
	ssp. *indica*		2x	
	ssp. *japonica*		2x	
Highbush blueberry	*Vaccinium corymbosum*	auto	2x, 4x, 6x	Colle et al. (2019)
Evergreen blueberry	*Vaccinium darrowii*	auto	2x, 4x*	J. Yu et al. (2021)
Cultivated potato	*Solanum tuberosum*	auto	4x	Sun et al. (2022)
Landrace/wild potato	*Solanum tuberosum*	auto	2x, 3x, 4x, 5x	Sun et al. (2022)
Maize	*Zea mays*	auto*	2x, 4x	Washburn et al. (2019)
Bermudagrass	*Cynodon dactylon*	auto, allo**	2x, 3x, 4x, 5x, 6x	Taliaferro et al. (2016)
Strawberry	*Fragaria x ananassa*	allo	8x	Sargent et al. (2009)
Watermelon	*Citrullus lanatus*	auto	2x, 3x, 4x	Wijesinghe et al. (2020)
Triticale***	*x* *Triticale*	allo	4x*, 6x*, 8x*	McGoverin et al. (2011)
Horseradish	*Armoracia rusticana*	allo	4x	Shen et al. (2023)
Sugarbeet	*Beta vulgaris *ssp. *vulgaris altissima* group	auto	2x, 3x*, 4x*	Sliwinska et al. (2005)
Oats	*Avene sativa*	allo	2x, 4x, 6x	He et al. (2024)
Greenland buttercup	*Ranunculus auricoms*	allo	2x, 4x, 6x	Ulum et al. (2020)
CULtivated sugarcane	*Saccharum officinarum*	auto, allo	8x	Bao et al. (2022)
Clover	*Trifolium*	allo	2x, 4x	Nadon And Jackson (2020)
Ryegrass	*Lolium perenne*	auto	2x, 4x	Rauf et al. (2021)
Soybean	*Glycine*	allo	2x, 4x	Sherman-Broyles et al. (2017)
Festulolium***	x*Festulolium*	allo	4x	Ghesquiere et al. (2010)
Coffee	*Coffea arabica*	allo	2x, 4x	Noir et al. (2004)
Cotton	*Gossypium*	allo	2x, 4x	Hu et al. (2021)
Quninoa	*Chenopodium quinoa* Wild	allo	4x	Maughan et al. (2019)
Tef	*Eragrostis tef*	allo	4x	Vanburean et al. (2020)

*denotes ploidy level that is not seen in wild populations

**denotes species with debated ploidy type

***denotes hybrid species

### Autopolyploids

Autopolyploids are often viewed as the same species as their progenitor. Examples include *M. sativa* ssp. *falcata*, that can be found at both the 2 × and 4 × levels, and arguably ssp. *varia* which results from ssp. *sativa* x ssp. *falcata* hybrids that can be 2 × or 4x (Small 2011). The *Vaccinium* (Blueberry) genus also provides an example of this. Taxonomically, the blueberry complex is made of multiple distinct species, some of which span multiple ploidy levels, such as *V. corymbosum* being 2x, 4x, or 6x. Differing ploidy levels are effectively genetically isolated because they are able to interbreed, but produce infertile interploidy offspring (Colle et al. 2019). Many autopolyploids have the distinctive genetic feature of multivalent pairing, where more than two homologous chromosomes may synapse, allowing for complex crossover events (Harlan And deWet 1975; Quiros 1982). Pairing pattern has historically been a defining characteristic of auto- vs allopolyploidy: multivalent pairing has been associated with autopolyploids. In contrast, bivalent-pairing is frequent in allopolyploids, although recent studies have indicated that chromosome pairing behavior is more of a continuum than a binary and may not be as specific to allo- or autopolyploids as previously thought (Nadon And Jackson 2020; Twyford et al. 2025). A study on the continuum of allo–autopolyploidy by Han et al. (2025) has shown that functionally, newly formed segmental allopolyploids can establish as autopolyploids. When two rice subspecies were crossed, *Oryza sativa* ssp. *japonica* and ssp. *indica*, and WGD was induced, within twelve generations of self-fertilization all chromosomes reached an average of 80% homozygosity, with some plants as high as 97%, making them functionally autopolyploids (Han et al. 2025). While this behavior may be limited to species that lack homeologous pairing suppressor genes (such as *Ph1* in wheat), it can provide a source of genetic diversity through high rates of recombination by creating synthetic autopolyploids with current elite cultivars (Al-Kaff et al. 2008; Han et al. 2025).

**Fig. 1:**
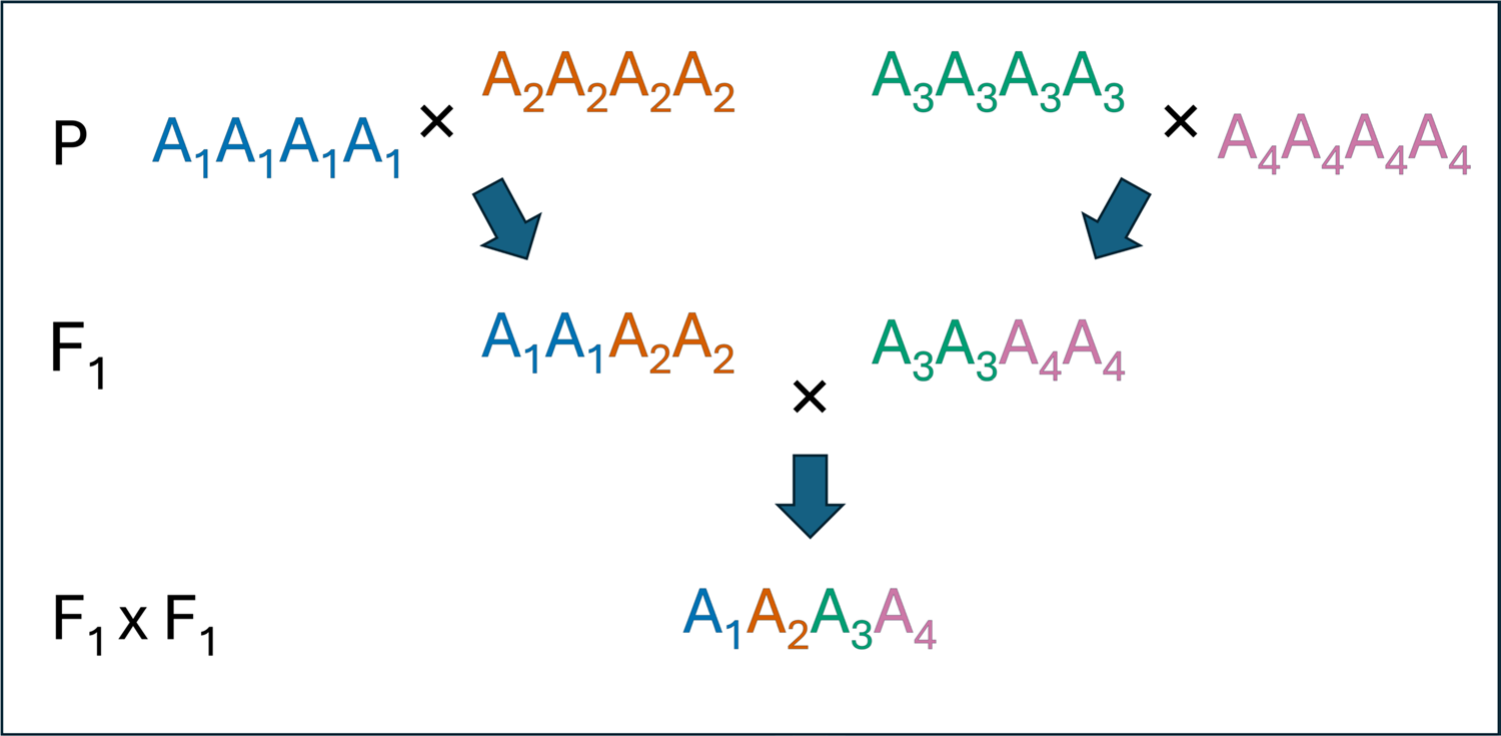
Simplified outline of a breeding scheme to exploit progressive heterosis. Parents are four highly homozygous individuals, each contributing a different allele, to produce offspring in the F1xF1 population that contain one copy of each allele (tetraallelic). Only the target offspring is shown, though numerous combinations of alleles are possible in the offspring of the F1xF1 cross that will not exhibit progressive heterosis to the same degree if they receive two identical alleles from either parent. This concept can also be extrapolated to the genome level, where receiving four unique/distinct haplotypes from 4 different homozygous parents could allow for many progressive heterosis interactions within the target offspring genotype

Alfalfa is an example of an autotetraploid where chromosomes mainly pair in bivalents with no preference between homologs (random pairing), resulting in typical tetrasomic inheritance (Quiros 1982; Rosellini et al. 2016). The interactions of multiple alleles in autopolyploid genomes can give rise to progressive heterosis (Washburn et al. 2019). Heterosis, also known as hybrid vigor, is the genetic phenomenon in which the hybrid offspring of two genetically diverse, often inbred, parents outperforms one or both (Chen 2013; Washburn And Birchler 2014). In turn, the inbred offspring of these hybrid heterotic plants lose vigor due to loss of heterozygosity (Chen 2013). Progressive heterosis takes this one step further, whereby double crossing four genetically distant parents, an autotetraploid can accumulate up to four different alleles at any locus, that is, a tetraallelic state (Parisod et al. 2010). A simplified outline of these crosses can be seen in Fig. 1. Progressive heterosis has been demonstrated in alfalfa, where autotetraploids from diverse genetic backgrounds were shown to be more vigorous than single-crossed diploids (Bingham et al. 1994; Brummer And Riday 2005). Progressive heterosis has also been experimentally observed in neopolyploid maize, where inbred lines were treated to induce tetraploidy and subsequently double-crossed, resulting in a substantial increase in above-ground biomass (Washburn et al. 2019).

### Allopolyploids

Allopolyploids are novel plant species that arise from combining two or more plant genomes into a single nucleus (Nadon And Jackson 2020). The progenitor species are usually closely related and from the same taxon (Mata et al. 2023). Allopolyploids can form through different mechanisms, the most common route being unreduced (2n) gametes that form due to meiotic dysfunction (Brownfield & Köhler 2010). Alternatively, normal, reduced gametes may combine to form a hybrid, and due to mitotic or meiotic abnormalities, both genomes spontaneously duplicate, resulting in allopolyploidy (Ansari et al. 2022; Nadon And Jackson 2020). Allopolyploidization as a result of interspecific hybridization followed by chromosome doubling was proposed in the early 1900 s (Winge 1917). This route has been used in research and breeding programs, obtaining convincing evidence of increased frequency of 2n gametes formation in interspecific hybrids (Ansari et al. 2022; Jauhar 2003; Tel-Zur et al. 2020; Zeng et al. 2020).

Allopolyploids can further combine to reach higher ploidy levels. Cultivated strawberry (*Fragaria* × *ananassa*), for example, is an allooctoploid (8x) combination of two allotetraploid species (Sargent et al. 2009). In the case of bread wheat, a free-threshing cultivated tetraploid species, thought to be a cross of *Triticum turgidum* subsp. *dioccoides* and *dicoccon* or wild and cultivated emmer (AABB) crossed with diploid *Aegilops tauschii* (DD) in the fertile crescent to form hexaploid *T. aestivum* (AABBDD), the cultivated bread wheat (Levy and Feldman 2022).

*Brassica* presents an interesting situation with the species called “The Triangle of U”. This group consists of an extended complex of diploid and allotetraploids that have undergone recurrent polyploidization. The triangle consists of diploids: *Brassica rapa (n* = *10*, AA*)*, *Brassica oleracea (n* = *9*, CC*)*, and *Brassica nigra (n* = *8*, BB*)*, and allotetraploids derived from all combinations of the diploids: *Brassica napus (n* = *19*, AACC*), Brassica juncea (n* = *18,* AABB*),* and *Brassica carinata (n* = *17*, BBCC*)* (Li et al. 2017).

While autopolyploid genomes generally form multivalents during prophase I, allopolyploids generally form bivalents within their genomes of origin, meaning that the two genomes remain functionally separate within the nucleus. However, species described as “segmental allopolyploids” deriving from very closely related progenitor species, or having undergone many homeologous exchanges, can exhibit multivalent pairing (Mason And Wendel 2020). An example is the allotetraploid *Arachis hypogea* or cultivated peanut (Bertioli et al. 2019). The progenitor species of cultivated peanut, *A. duranensis* and *A. ipanënsis*, are estimated to have diverged roughly 2.3–2.9 million years ago (Moretzsohn et al. 2013). This relatively recent divergence is further seen in the sequenced genomes of the diploid progenitors, showing high levels of similarity and the ability to interbreed to produce vigorous offspring. Similarity between the two sub-genomes within the tetraploid was also observed (Bertioli et al. 2016).

## Process of polyploidization

The cytological mechanisms associated with polyploidization have been well documented. It is generally accepted that the main routes of formation are a union of unreduced (2n) gametes, somatic chromosome doubling, or, less frequently, polyspermy (Bretagnolle And Thompson 1995; Mao et al. 2020; Palumbo et al. 2021). Selection pressure for inducing polyploidization is a highly debated topic. In some situations where polyploids reproduce asexually, it is theorized that this is an evolutionary adaptation to allow sexually reproducing diploids to generate clones of highly adapted polyploid genotypes that take advantage of ideal growing conditions. Species such as *Paspalum intermedium* have both naturally occurring diploid and autotetraploid populations with frequent polyploidization events associated with apomixis; research on these populations and their distribution indicates that environmental conditions trigger a switch in reproductive pathways to produce more polyploid individuals (Karunarathne et al. 2020). *Paspalum rufum*, another grass species with varying ploidy levels and recurrent autopolyploidization, has been genetically profiled to show that gene expression changes influenced by different environmental conditions during germ cell development may produce unreduced gametes and result in increased ploidy level in offspring (Siena et al. 2008; Soliman et al. 2021). This could indicate that one driving force of autopolyploidization is the ability to immediately exploit an optimum habitat by acquiring asexual reproduction triggered by WGD (Ulum et al. 2020). Other studies in the genus *Paspalum* have further explored this genus’s use of recurrent polyploidization events to increase survival rates depending on environmental conditions (Terzaroli et al. 2023).

The role of environmental stress and polyploidy is debated. Polyploids are often credited with having better stress tolerance than their diploid progenitor species (Van de Peer et al. 2021). Still, the view that polyploidy is more widespread in extreme environments (Rice et al 2019) has been challenged (Mata et al. 2023). In the *Ranunculus auricomus* complex, for example, asexual reproduction through apomixis is favored; however, when an extended photoperiod is applied to 2x, 4x, and 6 × individuals, the 2 × plants switch to sexual modes of reproduction (Ulum et al. 2020). Further studies on this species complex indicate that the polyploid populations are able to quench excess light better than diploids. The interpretation given by the authors is that diploids turn to sexual reproduction as a response to stress to induce new genetic variation, including the formation of new apomictic polyploids (Ulum et al. 2021). These advantages under stress may provide some explanation for autopolyploid establishment in nature.

Allopolyploid formation involves two or more progenitor species. Studying natural allopolyploids can be complicated because the progenitor species may be outcompeted by their polyploid offspring and either go extinct or take a divergent evolutionary route (Mata et al. 2023). On the other end, to establish, the neo-allopolyploid must either reproduce asexually, be self-compatible, or be able to hybridize with another neo-allopolyploid resulting from the same progenitor species.

Recurrent allopolyploidization is not widely observed in plant species because, unlike autopolyploids, the formation of allopolyploids requires both a mutation/deviation from normal sexual reproduction leading to the formation of 2n gametes and two parents of different species that can produce a viable offspring (Matsuoka et al. 2014). Instances of recurrent allopolyploidization were observed in areas where progenitor populations overlap. One example is the genus *Tragopogon*, where European species were introduced to Western North America in the early 1900 s, resulting in recurrent allopolyploid populations that subsequently out competed their diploid progenitors in the shared ranges (Soltis et al. 2004). A different pattern has been described for soybean, in which diploid wild relatives of the *Glycine* genus gave rise to reproductively isolated biological species rather than continual production of new tetraploids that could continue to breed with the established tetraploid populations (Sherman‐Broyles et al. 2017).

### Artificial polyploidization

Early in plant breeding history, it was observed that polyploidy could increase biomass (so called *gigas* effect, Müntzing 1936) and improve agronomic traits. This stimulated research into the possibility of artificially inducing WGD. Various methods or chemical treatments can induce polyploidy. Antimitotic agents such as colchicine, oryzalin, or trifluralin can be used to induce autopolyploidization. The most commonly used chemical, colchicine, is an anti-microtubule and antimitotic agent that causes the destabilization of microtubules, resulting in mitotic restitution and WGD (Eng And Ho 2019; Münzbergová 2017). Oryzalin and Trifluralin are dinitroaniline herbicides that act by binding to tubulin dimers to prevent microtubule formation during mitosis, though at a different site than colchicine (Lignowski And Scott 1972; Madon et al. 2005; Petersen et al. 2003). Application procedures vary though they generally use small propagules or young plants through spray or uptake in media to induce polyploidization, with varying success rates. These treatments may create ploidy chimeras, or any level of ploidy, so treated plants must be evaluated for chromosome number, desired traits, survival, and other mutations (Petersen et al. 2003). Artificial polyploids have been obtained and studied in innumerable species for both evolutionary studies and breeding purposes (see below).

## Effects of polyploidization on the genome

In studies of the effect of WGD, basic concepts have been defined and can be found in Box 1.

Polyploids present unique challenges for genomic studies. Changes in the genome can range from large-scale structural changes, such as deletions, insertions, or translocations, to expression level changes due to new gene interactions. These structural changes directly impact allele frequency and gene dosage. Tetraploid may have more than four possible alleles for a given gene, and due to structural changes, there may not be exactly four copies of each gene across the genome (Birchler And Veitia 2010). Allele frequency is defined, in genomics, as the frequency at which alleles appear in the genome, while gene dosage is the frequency at which a given gene appears in the genome. Different combinations of allele frequency or gene dosage may give different phenotypic traits. These structural changes allow for the long-term evolution of polyploids as genes diverge in function or accumulate mutations to develop new functionality (Birchler And Veitia 2010). In particular, allele frequency poses a major question when studying gene expression and functionality in autopolyploids. Duplication of the genome does not mean that a gene’s expression will always be doubled or increase, such that overexpression of homeologous genes responding to the same stimuli or interaction between related genes can disrupt pathways and cell function (Yu et al. 2023).

Studies on the impacts of WGD can be complicated due to the nuance of what “short term” means in the context of evolution and the cyclic nature of polyploid formation. The diploidization cycle (WGD followed by diploidization, followed by a new WGD, and so on) in plants is theorized to be an evolutionary mechanism that, over thousands, if not millions, of years, promotes new genetic diversity and speciation (Qiao et al. 2019). This review focuses on “immediate” impacts of WGD in non-established populations (first 1–3 generations after a WGD) compared to structural changes in fully established populations.

Box 1: A summary of commonly used terms in WGD studies

**Table:** 

Genome dominance describes nonequivalence of two or more sub genomes regarding overall gene loss following allopolyploid formation (Cheng et al. 2018)
Expression level dominance focuses on the total expression of a duplicated gene pair, and if the majority of genes share the same “dominant parent” that allopolyploid is considered to show “genome wide expression level dominance (Hu And Wendel 2019)
Homeolog expression bias in favor of one genome or another can occur in allopolyploids; it is defined as an unequal expression of two or more duplicated copies of a given gene, which can be evaluated from single genes to the entire genome (Hu And Wendel 2019; Cenci et al. 2019; Chelaifa, Monnier, and Ainouche 2010)
Additive or nonadditive expression levels refer to the total expression of all homoeologous or homologous copies in relation to the parental diploid averages, so that additive expression means that parental genes or genomes are equally expressed and nonadditive expression indicates an imbalance (Hu And Wendel 2019). This expression bias may lead to overall structural changes as mutations accumulate in the less expressed sub genome and portions of it may be lost (Cenci et al. 2019)
Diploidization is a long term outcome of WGD, whereby polyploids undergo high levels of mutation (Parisod et al. 2010), silencing and loss of duplicated genes (Wu, Han, and Jiao 2020)

### Autopolyploids

Cytologically, the most distinct difference between allopolyploids and autopolyploids is the frequency of multivalent pairings during meiosis (Nadon And Jackson 2020). This leads to a higher crossover; however, frequency varies across species (Parisod et al. 2010). Chromosomal mutations have been shown to create entire lineages within autopolyploid species. An example is the Draper variety of tetraploid blueberry, where translocations were noted on chromosomes 6 and 10 that were unique to this cultivar compared to others reviewed in the study (Mengist et al. 2022). A similar situation was described in *Musa acuminata* (Banana) subspecies, where a recent study identified six large reciprocal translocations present across the different subspecies, possibly giving rise to their differentiation (Martin et al. 2020).

Autopolyploidy may further provide an evolutionary path forward due to the presence of duplicated genes. The duplicated genes can compound effects, but they also can diverge without major consequence to the plant due to genetic redundancy (Adams And Wendel 2005). This divergence from their original functions can be categorized as either neo- or sub-functionalization. *Neofunctionalization* is defined as the acquisition of a new function by one of the duplicated genes, and is often the expected outcome of gene duplication, providing a new evolutionary path (Birchler And Yang 2022). Alternatively, the duplicate genes may split their original function, resulting in *subfunctionalization,* where the two members of the duplicated gene may become expressed in different tissues or developmental phases. In this case, both “new” genes are required for the organism to function (Birchler And Yang 2022). Divergence of genes is not unique to autopolyploids as allopolyploids have homeologous genes from the parental species, which can also diverge over time.

While some genes will diverge in function, others will undergo a process of *non-functionalization* if there is no selection pressure on them and they can accumulate random mutations (Cheng et al. 2018). This is often the case when allelic dosage does not require more than two copies of a gene to reach the needed transcriptional threshold to meet plant functional needs, though sometimes mutations will produce nonfunctional proteins that are not deleterious to plant health (Adams And Wendel 2005). This loss of function transforms the affected genes into pseudogenes, which are areas of non-functional DNA within the genome that resemble genes (Roulin et al. 2013). These pseudogenes can be lost over time since their deletion will not impact plant viability, and are a way to estimate the age of a polyploid species (Cheng et al. 2018).

Autopolyploids are likely to have multiple alleles for a given gene, resulting in complex and challenging to predict allelic interactions (Blischak et al. 2015), as mentioned above about progressive heterosis. This presents an issue for newly formed autopolyploids, because when the genome doubles, the possibility for accumulating deleterious alleles also increases (Yu et al. 2023). Depending on the allele, the dosage required for fatality may change with the increased ploidy level (Birchler And Yang 2022). This dosage change may result in genetic bottlenecks due to segregation distortion with genes closely linked to others that contain deleterious alleles if a low dosage is still fatal (Wu et al. 2001). Conversely, the presence of multiple non-deleterious alleles may be able to compensate for a condition that was fatal in the diploid progenitors (Soltis and Soltis 2000). Allelic diversity can compensate for some loss of function and allows for divergent mutations, where alleles can mutate to gain new functionality (Yu et al. 2023).

### Allopolyploids

Bread wheat represents a well-studied allopolyploid with bivalent-pairing within each of the three subgenomes (Da Ines And White 2015). *T. aestivum* is one of many allopolyploids from the *Triticum* and *Aegilops* evolutionary complex, both genera having high levels of allotetraploidy and allohexaploidy within themselves and their hybrids (Matsuoka et al. 2014). This evolutionary complex provides a clear model for understanding subgenome dominance patterns (Feldman et al. 2012). Expression level dominance of the A subgenome became subgenome dominance as gene retention frequency shifted in favor of the A subgenome compared to the other(s) over time (Alger and Edger 2020; Hu And Wendel 2019). The dominant “A” subgenome is shared across the polyploid *Triticum* species within the cluster, and studies have shown that it dominates for contribution to morphological traits while other subgenomes are responsible for local ecological adaptation (Chelaifa et al. 2013; Feldman et al. 2012). Subgenome expression bias and subgenome dominance are still not completely understood. The current thought is that bias and dominance are ways for allopolyploids to mitigate interference between homeologous genes or gene pathways. The dominant genome will “recruit” beneficial genes from the subgenomes to enhance pathways and survival while silencing or diverging genes with duplicate functions (An et al. 2024; Feldman et al. 2012).

#### Immediate impacts of WGD

Here, “short-term impacts” are defined as structural or expression level changes in polyploids formed within the last 300 years as per other studies (Edger et al. 2025). “Immediate impacts” are defined as changes within the first 2–3 generations of neopolyploids after the initial event. Neopolyploids can occur in one of two ways: a new WGD event resulting in a new species, or a recurrent WGD event within a species complex where some polyploids are established but new neopolyploids are actively formed by extant progenitor populations (Siena et al. 2008; Soltis and Soltis 1999). Specific new polyploid species are challenging to pinpoint and study; however, recurrent polyploids give an ideal model for determining immediate impacts of WGD by comparing immediate neopolyploids to established polyploids that span the spectrum of short-term to long-term. Two well-studied examples of recurrent polyploidization are seen in the *Medicago sativa* complex and the *Musa* (banana) complex (Simmonds And Shepherd 1955; Small 2011).

Within the *Musa* complex, polyploids are still occurring due to subspecies hybridization. Still, established subspecies can be tracked by distinctive translocation events that have improved performance in a subspecies’ natural range (Martin et al. 2020). In this situation, the number of translocation events can be compared to determine WGD age. Cultivated triploid banana (mostly allo- or autotriploids) gives a unique model for studying impacts of genome duplication in a clonally propagated species, since clones are maintained for many years but are still the “first generation” of polyploids Li et al. (2024), though established and recurrent tetraploids are also studied (Amah et al. 2019). Most cultivated banana varieties have been established in the last 100 years, though their genetic material is much older (Heslop-Harrison And Schwarzacher 2007). In vitro polyploidization of 2 × bananas has shown that immediate effects in the first generation of neoautotetraploids may not be agronomically advantageous (Amah et al. 2019). In this study, neoautotetraploid plants had larger, weaker leaves prone to breakage, and fewer, though larger, fruits(Amah et al. 2019). Time to flowering was longer in neoautotetraploids; however, maturation after flowering was faster.

In another work, using a combination of agronomic traits and molecular markers for assessing variability in neoautotetraploid banana (De Carvalho Santos et al. 2019), the team was able to show that the WGD event itself can introduce new genetic and phenotypic diversity. This study further examined the meiotic patterns of these neoautotetraploids, showing that not all could produce large amounts of viable gametes (De Carvalho Santos et al. 2019).

(Li et al. 2024) described transcriptional and structural subgenome dominance of the *M. acuminata* ssp. *banksii* in allotriploid production cultivars where the other subgenomes originated from different subspecies, with the dominant genome retaining more ancestral genes based on k-mer analysis. In these triploids, the other subgenomes were contributed by ssp. *malaccensis* and *zebrina*. The triploid *M. acuminata* is infertile and propagated through cloning, so each of these represents a short-term response to WGD. This immediate dominance pattern is seen in other crops, such as strawberries, showing dominance to subgenome A (Fang et al. 2024), and the wheat complex, where often the A subgenome emerges as dominant when present (Feldman et al. 2012).

Within the *M. sativa* complex, recurrent polyploidization allows diploid populations to be used in tetraploid alfalfa breeding programs (Small 2011). The main species included within the *Medicago sativa* complex are *M. sativa* ssp. *caerulea* (2x), *M. sativa* ssp. *falcata* (2 × and 4x), *M. sativa* ssp. x *hemicycla* (2x), *M. sativa* ssp. *sativa* (4x), and *M. sativa* ssp. x *varia* (4x) (Inostroza et al. 2021; Şakiroğlu and İlhan 2021; Small 2011). The aforementioned subspecies do not encompass the entire complex, and an exhaustive list can be found in (Small 2011). Previous studies examining chloroplast haplotypes across the complex showed that while 4 × *M. sativa* ssp. *sativa* and 4 × *M. sativa* ssp. *falcata* are thought to originate from similar pools of diploids, the 4 × *falcata* shared chloroplast DNA sequence with *M. prostrata* instead of diploid *falcata,* indicating the possibility of at least two separate WGD events in the recent history of the complex (Havananda et al. 2011). This shows that tracking the origins of polyploids can be difficult.

Studies on wild neotetraploid alfalfa have yielded some insight into these immediate impacts. It has been demonstrated that 4 × *M. sativa* has increased cell size, leaf size, and biomass production compared to 2 × full siblings (Rosellini et al. 2016). Further study has shown that WGD directly impacts the transcriptome in these plants (Santoro et al. 2025). Specifically, in this study, it was observed that the male parent, a ssp*. falcata x caerulea* hybrid showed expression dominance over the female ssp. *falcata* parent (Santoro et al. 2025). While this sample size is too small to determine if the *caerulea subgenome* will always dominate over the *falcata subgenome*, it does show that expression dominance can be observed immediately after a WGD event. Chemically induced tetraploids from *M. truncatula* and *M. scutellata* showed similar morphological changes to newly produced neotetraploid *M. sativa* (Innes et al. 2021). While most of the current research focuses on comparing diploids to established tetraploids, the *M. sativa* complex provides a very useful genomic resource to study the immediate impacts of WGD.

#### Impacts of WGD on the genomes of established polyploids

The impacts of WGD in established polyploids have been thoroughly reviewed (Amah et al. 2019; Salman-Minkov et al. 2016; Twyford et al. 2025). Here, data available in the *M. sativa* complex are discussed. A study comparing a newly developed reference genome for a wild 2 × *M. sativa* ssp. *caerulea* against published 4 × *M. sativa* reference genomes found over 25,000 genes with copy number variations between the tested genotypes (Shi et al. 2024). This study found the 2 × and 4 × genomes were highly syntenic. However, consistent translocations were seen between chromosome 2 of the 2 × genotype to all haplotypes of chromosome 8 of the 4 × genotype, suggesting this translocation may predate the WGD event (Shi et al. 2024). This is also seen on 2 × Chromosome 6 to 4 × chromosome 2 and 2 × chromosome 3 to 4 × chromosome 1 (Shi et al. 2024). Some translocations were identified in only some of the 4 × haplotypes, such as the translocation from 2 × chromosome 5 to 4 × chromosome 1 in two haplotypes, which could indicate an area of post-WGD structural change (Shi et al. 2024). While this gives no means an exhaustive picture of the long-term impact of WGD, it highlights the advances in technologies that have made these types of studies feasible on a genotype-by-genotype basis and how this work will continue to be more accessible.

## Genome sequencing in polyploid crops

### Overview

When attempting to sequence entire chromosomes to discern haplotypes, assembly can be challenging because cross-over between haplotypes makes it difficult to distinguish true cross-over events from areas with high similarity between haplotypes (Zhang et al. 2019). Even with diploid species, shorter reads may fit into multiple locations within the assembly, posing a challenge in determining their true location, which exacerbates the increased haplotype count in polyploids (Zhang et al. 2019). While using reference genomes or data from related genotypes with high synteny can help with this, the hierarchical nature of genome assembly implies overlapping reads in multiple locations across multiple haplotypes can be difficult to anchor (Zhang et al. 2019). In cultivated potato, this issue has been noted and somewhat overcome in developing a haplotype-resolved genome using long-read technology and extensive SNP identification (Mari et al. 2022). Sugarcane Hi-C data has been used to anchor chromosomes to a haplotype physically (Bao et al. 2022; Zhang et al. 2019).

Allopolyploids contain genomes from different progenitor species, thus chromosomes preferentially pair with their homologs rather than their homeologs (Lloyd And Bomblies 2016). However, pairing between homeologs can occur and result in homeologous recombination, where portions of the genomes are mixed and must be disentangled and verified as cross-over events (Mason And Wendel 2020). Since the subgenomes are related, it can be challenging to determine if these crossover events are true events or areas of the genomes that are similar between the subgenomes (Mason And Wendel 2020).

Sequencing techniques and assembly tools have made great strides in recent years, allowing for advances in polyploid genomic research. Third generation, long-read sequencing techniques, such as the Pacific Biosciences’ single-molecule real-time sequencing (SMRT) and nanopore sequencing from Oxford Nanopore Technologies (ONT), allow for highly accurate long reads that can help reconstruct the entire chromosome sequence, telomere to telomere (T2T) (Lin et al. 2021; Roberts et al. 2013). These technologies produce highly accurate (99.8%) long high-fidelity (HiFi) reads with average lengths over 10 kb (Wenger et al. 2019). Moreover, techniques such as Hi-C allow for the study of chromatin in a 3D context by marking ligation junctions to explore the connections of genomic regions across chromosomes (Belton et al. 2012). Second-generation sequencing techniques, such as Illumina, are still widely used due to the low cost and high data yield of high-throughput short-read sequencing (Cox et al. 2010).

**Fig. 2:**
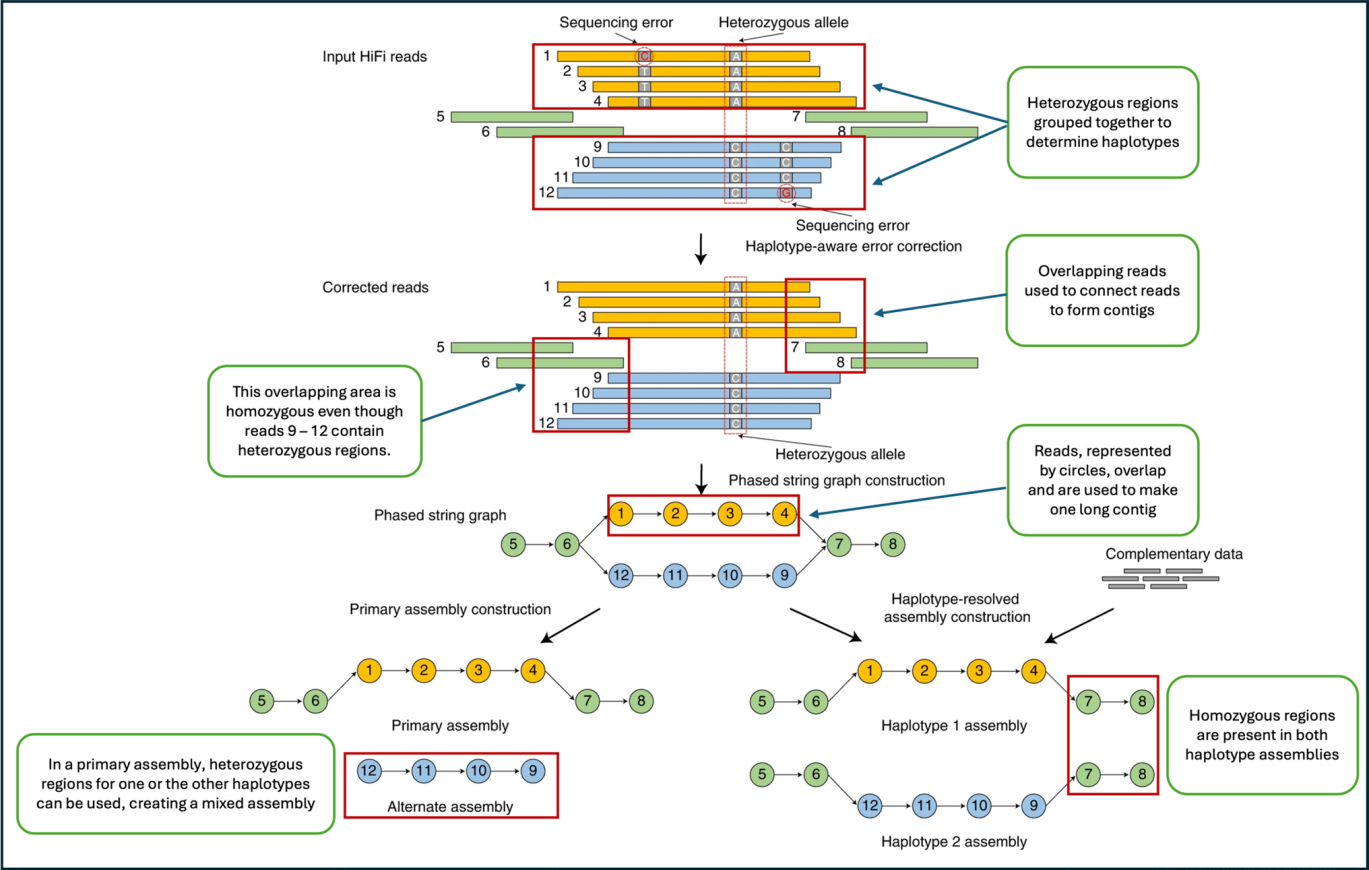
Graphic overview of Hifiasms genome assembly. Orange and blue bars represent reads with heterozygous alleles, while green bars represent homozygous regions. In the phased string, reads are aligned until a common point of overlap is found. Haplotype-aware error correction is used to distinguish heterozygous alleles from sequence errors. The phased assembly is built with the corrected reads. Hifiasm generates a completely phased assembly for each haplotype and an unphased primary assembly. This unphased primary assembly represents phased blocks (regions) that are resolvable with HiFi reads but does not preserve phasing information between highly heterozygous regions, creating a mixed assembly. Complementary data such as short reads or Hi-C data can be used to increase assembly coverage and haplotype phasing accuracy (modified and summarized from Cheng et al. 2021)

Assembly tools such as Hifiasm have been developed to handle HiFi data and have made it possible to produce highly accurate haplotype-resolved assemblies (Cheng et al. 2021). An outline of the Hifiasm algorithm from the original publication is provided in Fig. 2. Other tools, such as HiCanu, have been developed to utilize HiFi data from assemblers such as Canu, which was initially designed for second-generation assembly (Nurk et al. 2020). After initial assembly, the resulting contigs can be cleaned and aligned with various tools depending on the data (long reads, short reads, Hi-C) and resources (reference genomes, parental/related assemblies) available. While these advances have streamlined highly accurate diploid genome assembly, polyploids continue to pose challenges and often require combinations and creative use of multiple sequencing technologies and assembly tools (Wang et al. 2023). The following sections will examine some novel studies on auto- and allo-polyploids.

### Autopolyploids

Autopolyploids generally differ from allopolyploids in that a diploid variant is often available for alignment (Chen et al. 2020). However, this method is not always effective as mutations and chromosomal structural changes may disrupt alignment. This is notable in the *Medicago* genus. Tetraploid alfalfa genome sequences were often aligned on the *M. truncatula* genome, a diploid, homozygous relative, before the publishing of the tetraploid alfalfa genome in 2020 (Chen et al. 2020; Shen et al. 2020). Due to the genome of *M. truncatula* being smaller than *M. sativa* there were genes that were difficult to map, and other methods of assembly had to be employed (Chen et al. 2020).

A haplotype phased reference genome has been created for autotetraploid *Vaccinium corymbosum* (highbush blueberry) using second-generation sequencing technology (Colle et al. 2019). While it was possible to assemble and annotate this reference genome, it still remains difficult to develop cost and time effective high-quality haplotype-resolved genomes for studies in blueberry and generally autopolyploids.

Many studies are focusing on development of a high-quality diploid genome sequences as a milestone to sequencing tetraploid genomes. This has been done in both wild relatives of alfalfa (*M. sativa* ssp*. caerulea)* (Shi et al. 2024) and evergreen blueberry (*V. darrowii*) (Yu et al. 2021) using third generation long-read technology. These high-quality diploid genomes provide needed insight into development of higher ploidy references.

**Fig. 3:**
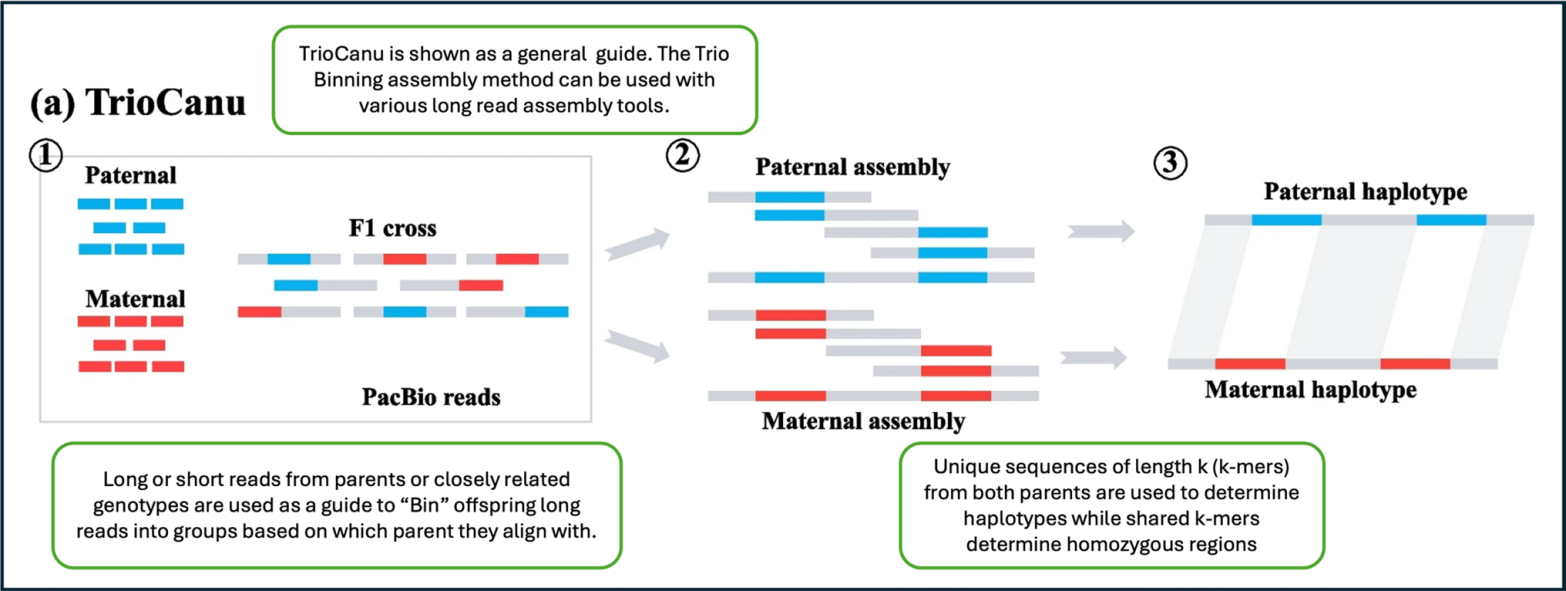
Modified from Zhu, K., Li. et al. 2024. Graphic originally used to depict Trio-binning with TrioCanu but used as a general guide for Trio-binned assembly methods here. Reads from parental or closely related genotypes are used to determine haplotypes during the genome assembly process to increase accuracy when linking heterozygous regions across the genome into their unique haplotypes. Before contigs are assembled, reads are “binned” into groups based on k-mer similarity to either parent genotype or determined to be homozygous and shared between both haplotypes. Contigs for each haplotype are assembled only using their respective “bin” and homozygous shared reads

Tools are also being developed for polyploid assembly. The Hifiasm tool mentioned above was originally developed for diploid genome assembly, but recently it has been used to assemble autotetraploid genomes. A notable example is the combination of HiFi and Hi-C data to create a haplotype-resolved potato genome (Sun et al. 2022). Trio-binning, an assembly method that uses data from parents to sort haplotypes in the offspring, has been a useful tool in developing autopolyploid reference genomes when parental genotypes or related diploids are available and can be seen in Fig. 3 (Koren et al. 2018). Trio-binning can be used with multiple genome assemblers, most notably Hifiasm in Trio-Binning mode or TrioCanu, a variation of the Canu assembly tool adapted to use a Trio-Binned assembly method (Koren et al. 2018).

### Allopolyploids

Allopolyploids present a challenge for sequencing because two or more genomes have to be parsed out that often have homology. Since progenitor species of an allopolyploid are usually related, there can be regions that are highly conserved between the genomes (Schiavinato et al. 2021). Templates from extant progenitor species can be used, but if the allopolyploid is long established there may be chromosomal structural shift, a difference in chromosome number, or major mutations that impact assembly accuracy (Garg et al. 2024; Shen et al. 2023). In some cases, no progenitor genome may be available, such as in *Eragrostis tef* or *Armoracia rusticana* (Horseradish) (Barker et al. 2016; Shen et al. 2023; VanBuren et al. 2020).

Peanuts and wheat provide good examples of allopolyploid species where innovative approaches have given rise to high-quality reference genomes. As mentioned previously, the progenitor species of cultivated peanut diverged evolutionarily recently (Bertioli et al. 2016). Cultivated peanut is a segmental allopolyploid, so cross-over between genomes is higher than in a bivalent-pairing allopolyploid, and this further presents a challenge when developing a reference genome because of the shared ancestry between the subgenomes (Bertioli et al. 2013, 2019). These studies used HiFi long-read data and Hi-C scaffolding to create a complete chromosome-scale assembly and to gain insight into recombination between the subgenomes. It was found that subgenome A lines up nearly perfectly with *A. duranensis* and subgenome B lines up with *A. ipaensis* aside from mutations that are present in all surveyed cultivars of cultivated peanut (Bertioli et al. 2016, 2019). It was previously noted that the highly repetitive sequences in the shared lineage of progenitor species made it difficult to separate the two genomes, so mapping mutations within the individual genomes and similarity to diploid progenitors helped overcome this limitation (Bertioli et al. 2013).

Wheat is a model for studying bivalent-pairing allopolyploids because there are multiple instances of polyploidization with well-known extant progenitor species (Matsuoka et al. 2014). The *Pairing Homeologous* or Ph1 gene has been well-studied across wheat subspecies, and is well conserved (Al-Kaff et al. 2008). This gene is responsible for preventing pairing between homeologous chromosomes, essentially keeping sub-genomes separate during meiosis and providing a partial barrier to intergenomic cross-over events (Griffiths et al. 2006), contributing to the stability of wheat and the diversification through allopolyploidization (Evans et al. 2022). Because of this stability, the International Wheat Genome Sequencing Consortium was able to make a high-quality reference genome with detailed sub-genome analysis using short-read sequencing technology (The International Wheat Genome Sequencing Consortium (IWGSC) Appels et al. 2018). This study was built off previous work that took a shotgun approach to studying this complex genome, where the diversity of the sub-genomes allowed for the development of an extensive reference genome for the time (Brenchley et al. 2012). While both of these projects were able to create detailed references, they were time consuming, computationally complex, and not feasible for crops that are not backed with the same resources as wheat.

In the early 2020 s, studies began to use long-read sequencing for wheat, most notably the Oxford Nanopore Technology (ONT) system was used to verify shorter reads, one of the earliest polyploid studies using third generation sequencing (Walkowiak et al. 2020). Shortly after, PacBio HiFi reads were used to construct an entire haplotype-resolved genome of cultivar Fielder, important in genetic engineering and gene editing studies (Sato et al. 2021). While challenges have persisted in dealing with highly repeated regions between the genomes, it is clear that the differentiation in sub-genomes, and agricultural value, has allowed wheat to be a pioneering species for polyploid genomic research.

## Polyploids in agriculture

Many important agricultural crops are polyploids, including: alfalfa, banana, coffee, cotton, oats, peanut, potato, quinoa, strawberry, sugar beet, wheat (He et al. 2024; Hilu 1993; Hu et al. 2021; Maughan et al. 2019; Nadon And Jackson 2020; Noir et al. 2004). Most polyploids had already undergone WGD and establishment before domestication (Akagi et al. 2022). Plants with increased ploidy levels can outperform their diploid progenitors with higher biomass yield, better stress tolerance, and more nutritional value (reviewed by Van de Peer et al. 2021). This increased performance may be due to the presence of more than two alleles per gene, allowing for more allelic and genic interactions that may increase phenotypic plasticity in the short term (Bingham et al. 1994; Chen 2013; Parisod et al. 2010; Tossi et al. 2022; Van de Peer et al. 2021).

The ability to artificially produce polyploids using colchicine or oryzalin has provided an interesting avenue for crop development (Gaeta And Chris Pires 2010). A few examples of successful artificial polyploids exist in both autopolyploids (banana, clover, ryegrass, sugar beet, watermelon) and allopolyploids (festulolium, triticale, citrus) (Aleza et al. 2009; Ghesquière et al. 2010; Rauf et al. 2021; Sliwinska And Lukaszewska 2005).

Triticale is taken as the best example of successful creation of a new polyploid species. It was generated by crossing wheat and rye, then treating the resulting offspring with colchicine (Zillinsky 1974). The experiments resulting in triticale started in the late 1800 s; however, it was not until the 1930 s that colchicine was used to double the chromosomes of the sterile *Triticum* x *Secale* hybrids to create viable 6 × and 8 × specimens (Ammar et al. 2004; Zillinsky 1974). In the 1960 s, breeding programs began to focus on introducing advantageous genes from rye and wheat to improve commercial viability (Mergoum et al. 2004a, b; Zillinsky 1974). It took roughly 15 years of breeding to make triticale commercially competitive against spring wheat, and improvements are still being made (Ammar et al. 2004; McGoverin et al. 2011; Mergoum and Gómez Macpherson 2004; Peña, 2004). While triticale highlights the successful creation of a new polyploid species, it also shows the limitations of this approach due to the intensive breeding and research needed to establish and make a new polyploid commercially viable.

Ornamental plants have been extensively improved by polyploidy (Niazian And Nalousi 2020).

While most polyploid crops are even number ploidy levels, species, such as banana and watermelon, are grown at a triploid (3x) level to produce sterile, seedless commercial varieties that have increased value compared to seeded varieties, though their production and improvement can be challenging (Cenci et al. 2019; Wijesinghe et al. 2020).

## Polyploidy in plant breeding

The potential of WGD for developing new traits finds limitations because polyploids present peculiar breeding challenges. In polysomic polyploids, complex segregation ratios can increase the generations needed to get desired allele frequencies and complicate prediction-based breeding methods (Husband 2016). On the other end, when working with allopolyploids, the diversity associated with allopolyploid formation poses a challenge in their study and utilization in breeding, and understanding the interactions between the subgenomes is imperative (Giles And Brown 2006; Sargent et al. 2009).

WGD, as a breeding tool, allows the sourcing of genes from diploid wild relative species for polyploid crop improvement in pre-breeding programs. This is common practice in potato (Carputo 2003). Bread wheat is an example of an allopolyploid where the progenitor species have been used in breeding projects. Synthetic hexaploidy wheat (AABBD’D’) derived by crossing tetraploid durum wheat (AABB) with the *A. tauschii* (DD) with subsequent artificial chromosome doubling leads to a synthetic hexaploid that can be crossed with cultivated bread wheat (Rosyara et al. 2019). In this situation, the nomenclature D’D’ is used because of the genetic distinction observed between the current *A. tauschii* genomes and the ancestral population that originally hybridized with the AABB tetraploid to give rise to *T. aestivum* (Rosyara et al. 2019).

A particular avenue of interest in allopolyploid breeding is understanding “fixed heterozygosity” or “fixed heterosis”. This concept was introduced in 1976, though the first major studies were not conducted until 2005 (Abel et al. 2005; MacKey 1976). Fixed heterosis is the idea that interactions between the homeologous genes of the different genomes in an allopolyploid can produce a positive heterotic reaction and contribute to the overall success of allopolyploids (Abel et al. 2005). It has been demonstrated in an allopolyploidy hybrid of *Arabidopsis thaliana* and *Arabidopsis arenosa* that the *FRI* (FRIGIDA) genes of the two subgenomes compound with each other resulting in extremely late flowering time (Wang et al. 2006). While these types of interactions have potential, the difficulty of clearly linking these physiological changes to fixed heterozygosity is a major limiting factor in implementing them as a breeding strategy (Soltis et al. 2014).

Some auto- and allopolyploids have various methods of clonal reproduction, that alleviates the complexity of polyploid breeding, of note are bananas (vegetative suckers and tissue culture), potatoes (sprouted tubers), and strawberries (runners) (Hummer And Hancock 2009; Jansky And Spooner 2018; Suman 2017; Tumuhimbise And Talengera 2018). These species represent polyploids where elite genotypes are relatively easy to maintain and market. Adversely, the clonal nature of some polyploids can also complicate breeding programs. Bermudagrass (debated on if it is an auto- or allo- polyploid), breeding typically focusses on the development of F1 hybrid plants that are then maintained through clonal propagation, but due to self-incompatibility plants must be outcrosses and bred as a population making breeding for specific traits difficult (Taliaferro et al. 2016).

Cultivated alfalfa is an obligate outcrossing autopolyploid and cannot be clonally propagated for commercial use. This can make it very difficult to fix and maintain traits of interest within a breeding program. Cultivated alfalfa can display heterosis when some genotypes are hybridized, but maintaining this heterotic gain within a population can be difficult (Annicchiarico et al. 2017).

Studying the process of polyploidization in autopolyploids is of interest to plant breeders because (wild) diploid germplasm may have desired genes or alleles that are not present in cultivated tetraploid germplasm (Li et al. 2011). This creates an opportunity for choosing genes of interest in diploid germplasm for introduction into a tetraploid breeding project that may be relatively easier than it is in allopolyploids. The *M. sativa* complex presents an interesting case study for understanding the immediate impacts of WGD in an autotetraploid model. A study looked at the impact of WGD using a progeny of *M. sativa* derived from two spontaneous meiotic mutants that when crossed produced both tetraploid and diploid offspring (Rosellini et al. 2016). This progeny is now used in genomic studies, taking advantage of newly available long-read sequencing methods to explore the short-term genomic events associated with WGD.

## Conclusions

Better understanding polyploids not only provides new scientific knowledge, but can also unlocks the use of new, diverse germplasm for crop improvement in the face of growing pressure on agriculture to produce more high-quality food with less environmental impact. Most diploid progenitors of polyploid crop species are wild and more adapted to harsh conditions in their native and wild ranges. These germplasms provide a valuable source of genetic material for adapting crop varieties to conditions, such as drought and salt, in the face of a changing climate (Dempewolf et al. 2014; Humphries et al. 2021; Inostroza et al. 2021). Species with large natural ranges, such as the *Triticum* x *Aegilops* complex, *M. sativa* complex, and *M. acuminata* complex, have been studies to identify useful diploids, but further work is needed to streamline their application in breeding programs (De Carvalho Santos et al. 2019; Inostroza et al. 2021; Levy and Feldman 2022).

High-quality reference genomes and more cost-effective sequencing technologies have made in depth genomic studies feasible for non-specialized researchers. A few years ago it was expensive, labor intensive and time consuming to create a haplotype-resolved polyploid assembly but the studies discussed in this review are paving the way for similar work in other species. We have seen the development of reference genomes in less studies species, such as Quinoa and Tef (Maughan et al. 2019; VanBuren et al. 2020), but we believe that these same technologies can be used to develop reference genomes of on non-cultivated germplasm of highly cultivated species. The wider use of wild germplasm genomes would further streamline their integration into cultivated breeding programs, given the large genomic differences between ploidy levels, established vs. neopolyploids, and non-cultivated vs. cultivated germplasm.

While polyploids as a whole represent a wide range of plant species, it is important to look for similarities between them when attempting new studies. Studies across autopolyploids face similar issues of highly similar haplotypes and novel assembly approaches can work between species. On a practical level, the possibility of using genomic studies of unreduced gamete production in alfalfa for production of triploid watermelon cultivars exists as a way to transfer knowledge between species that are not closely related (Palumbo et al. 2021; Wijesinghe et al. 2020). In-depth studies of repeated occurrences of allopolyploidization in wheat complex can provide insights and guidance on ways to study newer allopolyploids such as peanuts, informing ways to identify other related species that may be able to create further allopolyploids (Bertioli et al. 2019; Matsuoka et al. 2014). Polyploid genomics can provide a possible step forward for plant breeding of polyploid crops.

## Data Availability

Data sharing not applicable to this article as no datasets were generated or analyzed during the current study.
